# Supplementary material for: A Case Series Exploration of Multi-Regional Expression Heterogeneity in Triple-Negative Breast Cancer Patients
Source: Int J Mol Sci. 2022 Nov 1;23(21):13322. doi: 10.3390/ijms232113322 (PMC9655720; doi:10.3390/ijms232113322)
Supplement: Supplementary file 1 [file ijms-23-13322-s001.zip › Supplementary_ITH_IJMS_20221011.pdf]

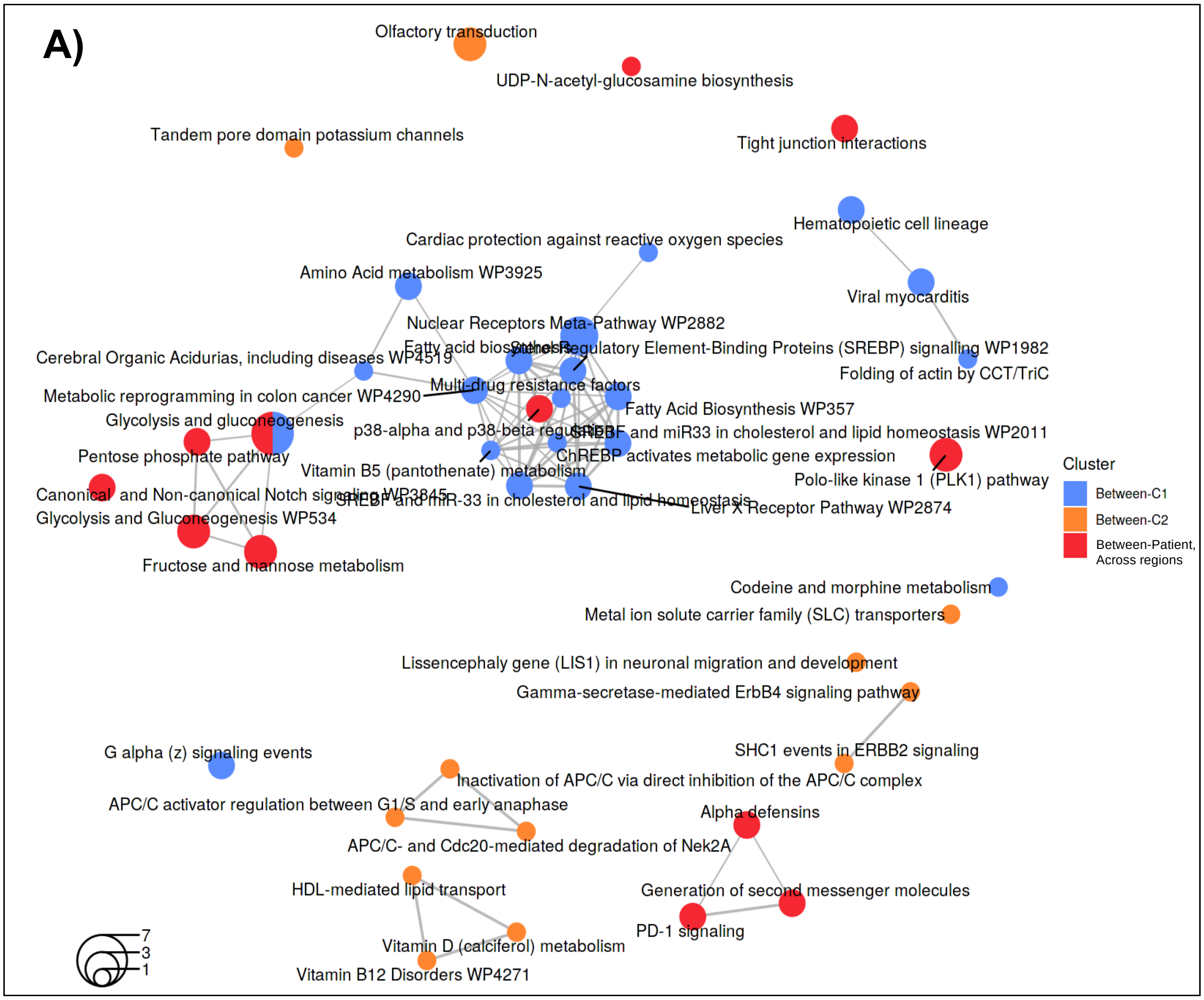
A)
Olfactory transduction
UDP-N-acetyl-glucosamine biosynthesis
Tandem pore domain potassium channels
Tight junction interactions
Hematopoietic cell lineage
Cardiac protection against reactive oxygen species
Amino Acid metabolism WP3925
Viral myocarditis
Nuclear Receptors Meta-Pathway WP2882
Cerebral Organic Acidurias, including diseases WP4519
Fatty acid biosynthesis
Sterol Regulatory Element-Binding Proteins (SREBP) signalling WP1982
Folding of actin by CCT/TriC
Multi-drug resistance factors
Metabolic reprogramming in colon cancer WP4290
Fatty Acid Biosynthesis WP357
Glycolysis and gluconeogenesis
p38-alpha and p38-beta regulation
SREBF and miR33 in cholesterol and lipid homeostasis WP2011
Pentose phosphate pathway
ChREBP activates metabolic gene expression
Vitamin B5 (pantothenate) metabolism
Polo-like kinase 1 (PLK1) pathway
Canonical and Non-canonical Notch signaling WP3845
SREBP and miR-33 in cholesterol and lipid homeostasis
Liver X Receptor Pathway WP2874
Glycolysis and Gluconeogenesis WP534
Fructose and mannose metabolism
Cluster
Between-C1
Between-C2
Between-Patient, Across regions
Codeine and morphine metabolism
Metal ion solute carrier family (SLC) transporters
Lissencephaly gene (LIS1) in neuronal migration and development
Gamma-secretase-mediated ErbB4 signaling pathway
G alpha (z) signaling events
SHC1 events in ERBB2 signaling
Inactivation of APC/C via direct inhibition of the APC/C complex
APC/C activator regulation between G1/S and early anaphase
Alpha defensins
APC/C- and Cdc20-mediated degradation of Nek2A
HDL-mediated lipid transport
Generation of second messenger molecules
PD-1 signaling
7
3
1
Vitamin D (calciferol) metabolism
Vitamin B12 Disorders WP4271

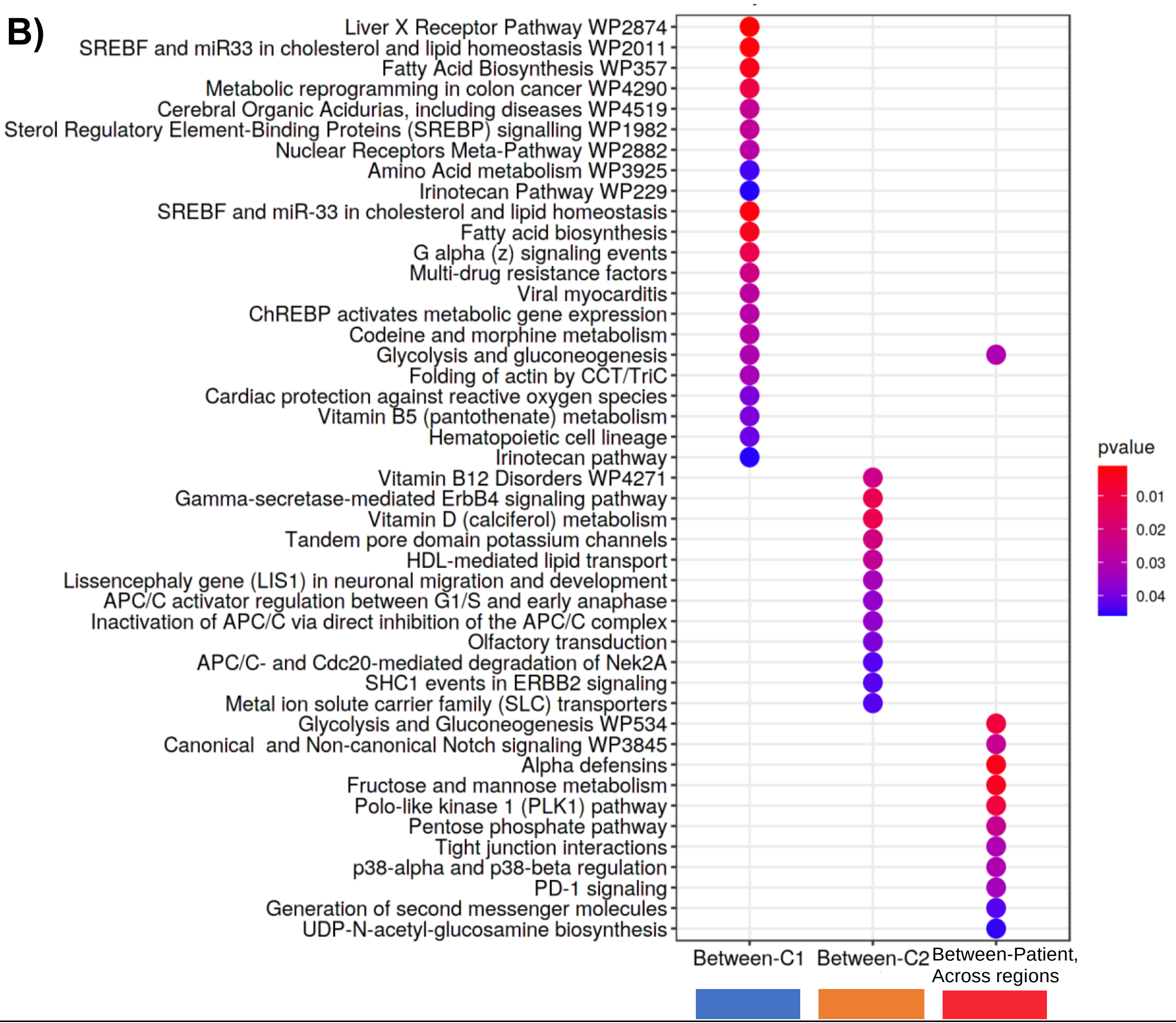

Supplementary Figure S1: Enrichment analysis showing distinct pathways characterizing the two main between-patient gene expression signatures and the within-patient signature. (A) Significantly enriched (p<0.05) WikiPathways and BioPlanet pathways among the 108 between-patient gene expression signatures (73 genes in blue, C1; 35 genes in orange, C2) and the within-patient signature (108 genes, red). **(B)** Dot plot of significantly (p<0.05) enriched pathways in each signature. The dots are color-coded according to p-value.

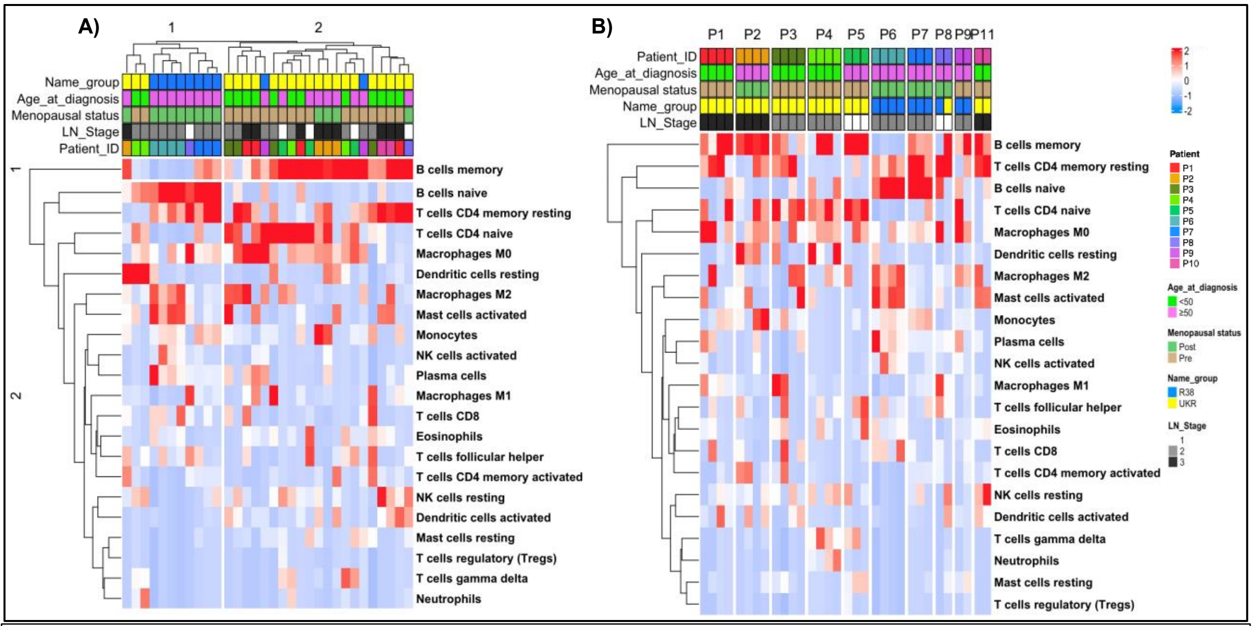

Supplementary Figure S2: Immune signatures differ among different regions of the same tumor. Heatmaps of gene expression-derived abundance scores (column scaled, complete linkage with Euclidean distance) for 22 immune cell types, with patient-level molecular and tumor characteristics highlighted. **(A)** Unsupervised clustering resulted in two sample clusters (denoted by 1 and 2). **(B)** Sample-supervised, patient-ordered clustering.

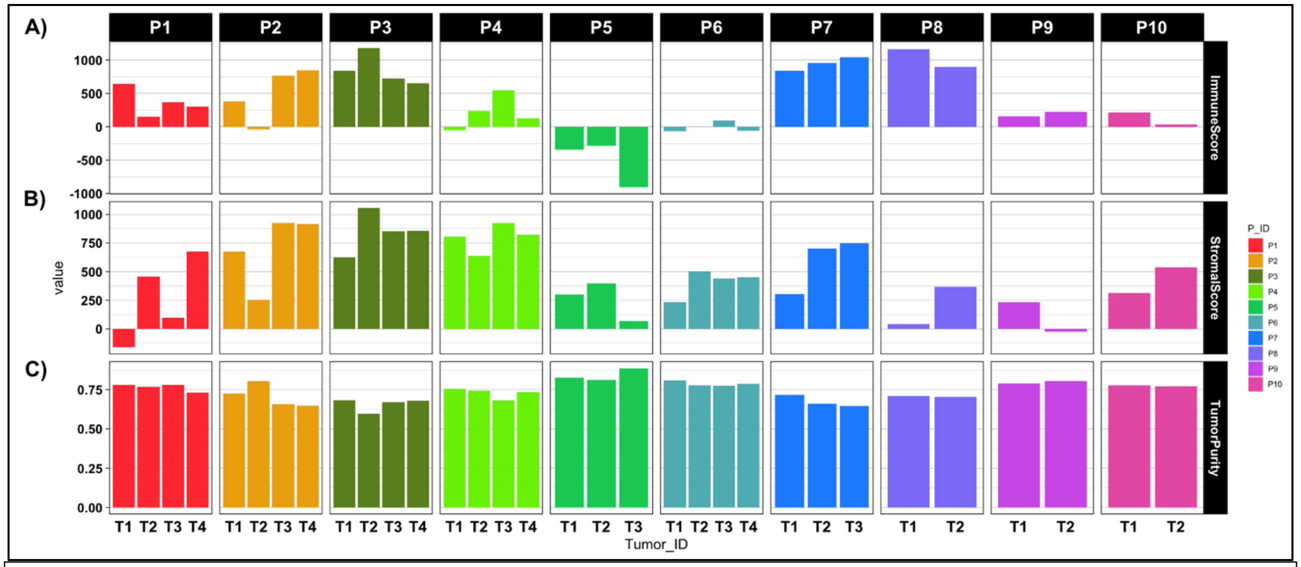

Supplementary Figure S3: Regional intratumoral heterogeneity in gene expression-derived immune, stromal, and tumor purity scores. Bar charts showing predicted gene expression-derived immune **(A),** stromal **(B)** and tumor purity **(C)** scores.

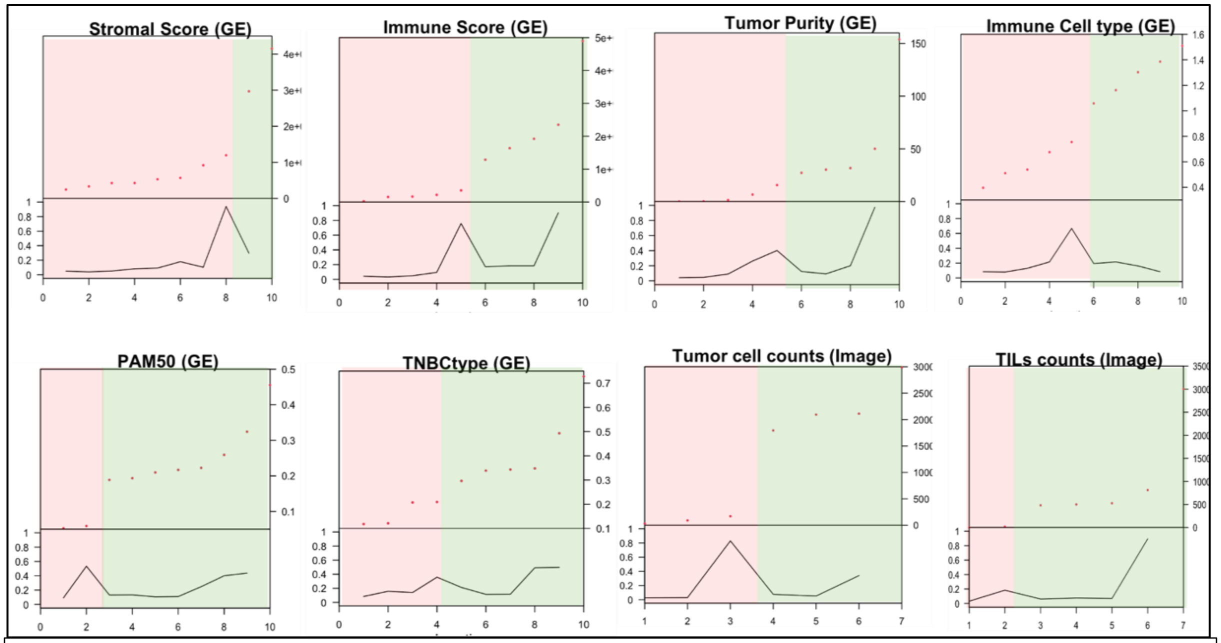

Supplementary Figure S4: Divergence analysis for regional variation in gene expression-derived and imaging-derived features. Plots of posterior means (above horizontal line) and posterior probabilities (below horizontal line) from Bayesian changepoint analysis applied to pairwise (among regions, within patients) Euclidean distances for each feature. Each red dot represents a patient. Patients to the right of the identified changepoint are categorized as divergent (green), and to the left, as convergent (pink).

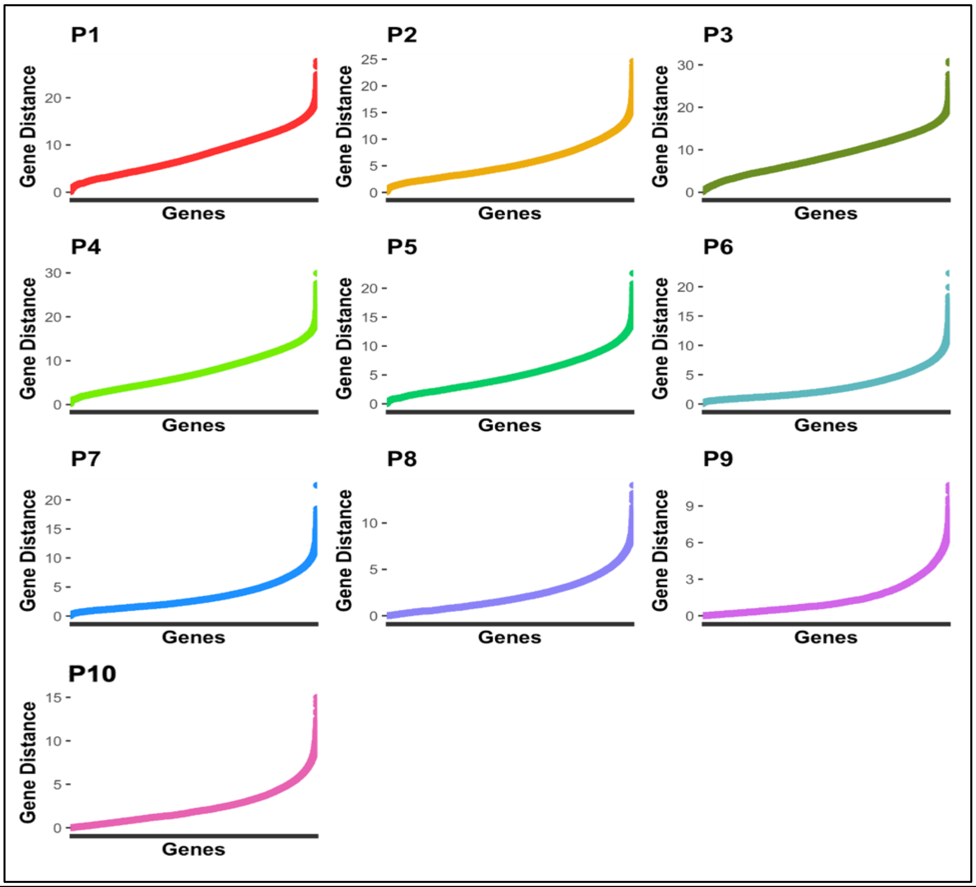

Supplementary Figure S5: Distributions of regional intratumoral heterogeneity in gene expression. Scatter plots of ordered individual gene-level expression differences based on pairwise (among regions) Euclidean distance (denoted by gene distance) for each patient.

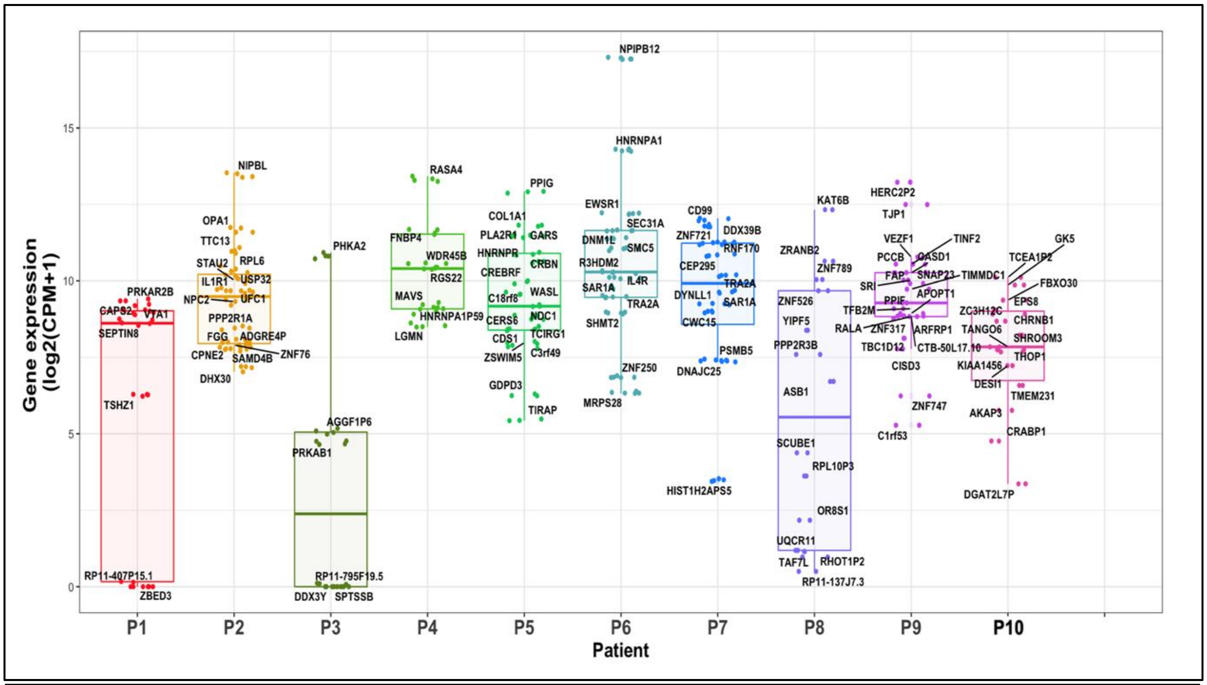

Supplementary Figure S6: The distribution of intratumoral region heterogenetiy low variability gene signatures by patient. The boxplots shows that low-variable gene signatures are mostly expressed among all regions in each patient, only with the exception of a few non-coding genes (e.g., RP11) in certain patients (P1, P2, P8).
